# Deep learning-based automatic facial symmetry scoring in peripheral facial palsy

**DOI:** 10.1038/s41598-025-17172-1

**Published:** 2025-08-27

**Authors:** Andreas Heinrich, Gerd Fabian Volk, Christian Dobel, Orlando Guntinas-Lichius

**Affiliations:** 1 Department of Radiology, Jena University Hospital – Friedrich Schiller University, Am Klinikum 1, 07747 Jena, Germany; 2 Department of Otorhinolaryngology, Jena University Hospital – Friedrich Schiller University, Am Klinikum 1, 07747 Jena, Germany; 3 Facial-Nerve-Center Jena, Jena University Hospital – Friedrich Schiller University, Am Klinikum 1, 07747 Jena, Germany; 4 Center for Rare Diseases, Jena University Hospital – Friedrich Schiller University, Am Klinikum 1, 07747 Jena, Germany

**Keywords:** Automated symmetry score, Deep learning, Facial paralysis, Image processing, Computer-assisted, Computational biology and bioinformatics, Diseases, Health care, Mathematics and computing, Medical research

## Abstract

Unilateral peripheral facial palsy (PFP) results in facial asymmetry and functional impairment, reducing quality of life. Accurate, objective assessment is vital for monitoring and rehabilitation. This study presents an automated method utilizes standardized 2D photographs to visualize facial dynamics using heatmaps and calculates an objective symmetry score, quantifying movement symmetry. Retrospective analysis included 405 facial datasets from 198 PFP patients. Images were processed using a deep learning-based facial landmark detection and an affine alignment algorithm. Heatmaps were generated from grayscale difference images, and symmetry scores calculated by comparing mirrored facial halves within a defined mask. Stennert movement scores were correlated with symmetry scores using Spearman’s rank correlation. The method was applied successfully to all datasets, with symmetry scores ranging from 0 to 0.99 (mean 0.85 ± 0.12), varying by expression level. Heatmaps highlighted asymmetries matching clinical findings. In 85% of cases, Stennert trends aligned with symmetry scores; 9% showed stable Stennert scores but changing symmetry scores, suggesting higher sensitivity. Significant negative correlations (r = − 0.32 to − 0.66, *p* < 0.0001) confirmed greater clinical severity corresponds to lower symmetry scores. In conclusion, the automated method provides an objective, reliable, and accessible tool for assessing facial symmetry in PFP, thereby improving clinical evaluation and facilitating precise rehabilitation monitoring.

**Supplementary Information:**

The online version contains supplementary material available at 10.1038/s41598-025-17172-1.

## Introduction

Peripheral facial palsy (PFP) substantially affects quality of life due to facial asymmetry, functional impairment, and psychosocial distress^1–4^, often complicated by facial synkinesis in the chronic stage of the disease^5^. Accurate assessment of facial muscle activity, which reflects the functional output of the facial nerve, is essential for monitoring recovery and guiding rehabilitation^6^. Improvement of facial symmetry is the primary goal of facial rehabilitation^7,8^. Although various clinical grading scales and questionnaires are available, these often rely on subjective facial grading, resulting in limited objectivity and interrater variability. Technological advances have enabled the development of computerized assessment methods providing objective and reproducible measurements. However, the clinical availability and validation of such automated tools remain limited, and no universally accepted gold standard has been established^9^. Many objective approaches require complex setups, high-cost equipment, or extensive manual input, restricting their feasibility for routine clinical use. This underlines the need for practical, accessible, and reliable methods to objectively evaluate facial symmetry and dynamic movements in patients with PFP.

This study aims to evaluate a new automated method using standardized photographic recordings of patients with PFP to visualize dynamic changes between neutral and expressive facial states through heatmaps and to derive an objective symmetry score that quantifies the uniformity of facial movements between the two sides of the face.

## Materials and methods

The study was approved by the local institutional review board (IRB) at Jena University Hospital (registration number 2019-1539-BO). Due to the retrospective nature of the investigation, written informed consent was waived by the IRB at Jena University Hospital. All methods were performed in accordance with the relevant guidelines and regulations, and the study was conducted in accordance with the Declaration of Helsinki.

### Study population and image acquisition

A retrospective analysis was performed on 518 datasets. Although no formal standardization criteria such as fixation devices or anatomical reference lines were applied, image acquisition followed a consistent protocol: All photographs were taken by a dedicated clinical photographer, with the patient seated in front of a uniform, neutral-colored paper backdrop. The photographer was seated directly opposite the patient, allowing for a reproducible frontal perspective. The camera was handheld to permit flexible adjustment of angle and height, while an approximately consistent distance was maintained across sessions. Two softboxes supplemented the camera flash to ensure even facial illumination. This setup provided sufficient consistency for comparative analysis under real-world clinical conditions.

The inclusion criteria required the availability of nine standardized facial photographs per patient. After applying these criteria, 405 datasets from 198 patients with unilateral PFP (94 female, 104 male) were included, with examinations conducted between March 26, 2008, and December 20, 2011. The age at examination ranged from 4 to 90 years (mean 53 ± 19 years). The age distribution of the total 405 datasets is as follows, grouped in 20-year intervals: 10 datasets aged 4–19 years, 118 datasets aged 20–39 years, 98 datasets aged 40–59 years, 159 datasets aged 60–79 years, and 20 datasets aged 80–90 years. Among these patients, 98 had at least two datasets acquired on different days during therapy. Details on the underlying etiologies and treatment can be found elsewhere.

For each patient, nine standardized facial photographs were taken, capturing the following expressions:

1. Neutral facial expression (reference)
2. Eyes gently closed
3. Eyes tightly closed
4. Frowning/forehead wrinkling
5. Nose wrinkling
6. Closed-mouth stretch
7. Mouth stretch with teeth showing
8. Lip pursing
9. Mouth corners down

Images were acquired primarily using Nikon DSLR cameras (mainly D90 and D100 models), with a smaller number from Canon and Sony devices. Flash was used in 309 cases, not used in 82, and the status was unknown in 14. Auto white balance was applied in 294 cases, manual in 97, and unknown in 14. Image resolutions varied widely, with the most common sizes being 1772 × 2362 pixels (189 cases), 709 × 1063 pixels (125 cases), and 712 × 1063 pixels (28 cases), ranging from 591 × 882 to 2632 × 3963 pixels.

### Image preprocessing and analysis

**Fig. 1:**
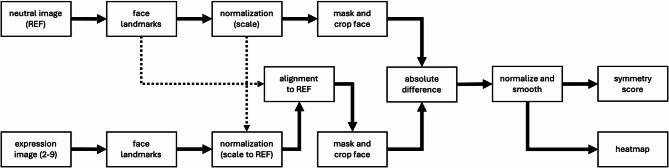
Processing steps using a neutral facial expression reference (REF) and an expression image.

The neutral facial expression image from the standardized facial photograph series served as the reference for automated comparison with expression images. In other words, the absolute difference is calculated between each expressive image and the individual’s neutral facial expression, which serves as the baseline. Subsequently, the symmetry score is computed from this difference image by comparing the left and right facial halves. This means that each symmetry score reflects deviations from the patient’s own neutral state, allowing assessment of dynamic changes rather than static asymmetries. Figure 1 illustrates the method’s processing workflow, with each step explained in more detail in this section.

**Fig. 2:**
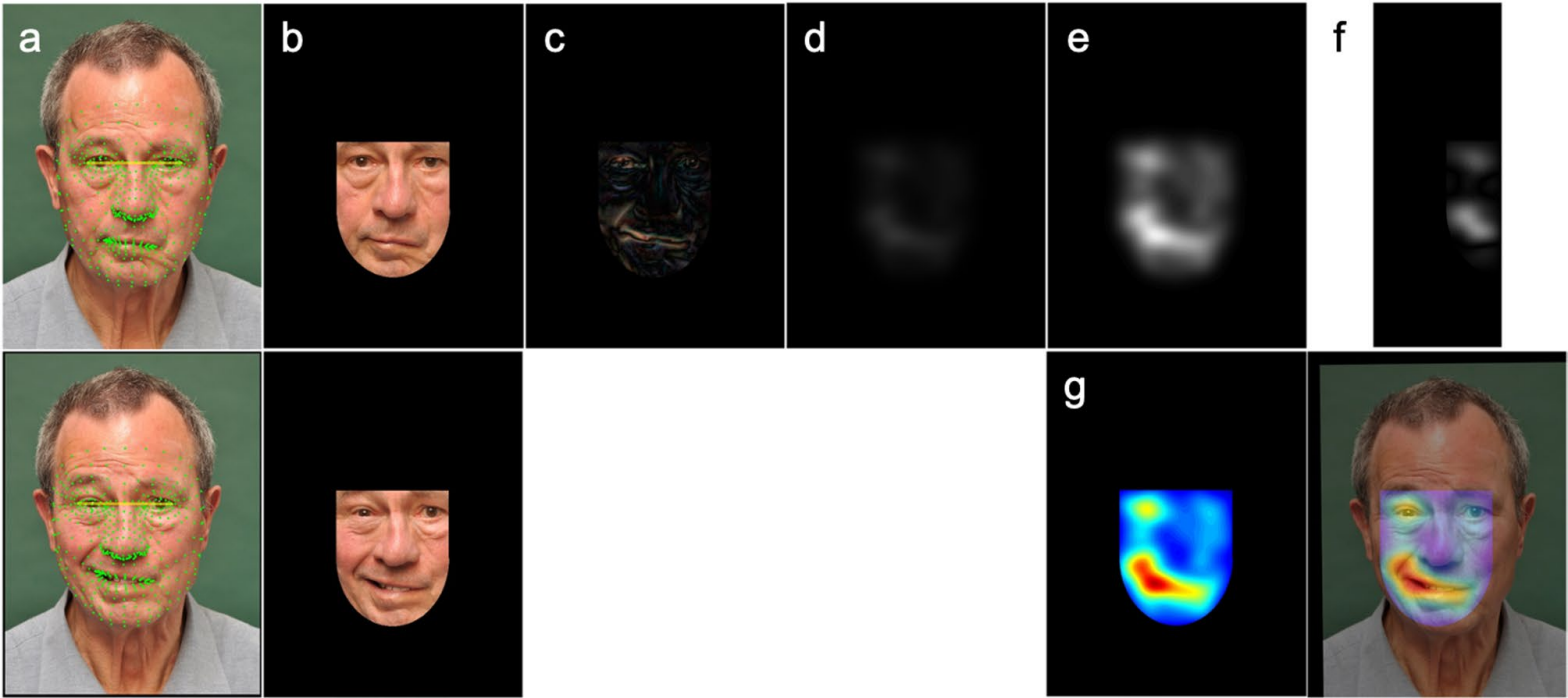
The image processing steps include (**a**) facial landmark detection of 478 points and uniform scaling so that the interocular distance equals 200 pixels; (**b**) alignment of the expression image to the neutral reference and application of a face mask isolating key facial regions; (**c**) calculation of the absolute difference between the neutral reference and aligned expression image; (**d**) Gaussian smoothing of the difference image; (**e**) scaling by a factor of 5 to normalize pixel intensities; (**f**) computation of the symmetry score based on the pixel differences between the left and the mirrored right side of the mask in (**e**); and (**g**) visualization of facial movements using a heatmap. Written informed consent for publication of these identifiable photographs was obtained from the patient.

Each image was processed in Python version 3.10.9 (Python Software Foundation^10^) using a deep learning-based facial landmark detection model implemented in MediaPipe (v0.10.21, Google Research^11^) with refined landmark prediction enabled, estimating 478 facial landmarks per image (see Fig. 2a). Subsequently, the Euclidean distance between the outer eye corners was used to uniformly scale all faces so that the interocular distance equaled 200 pixels. Expression images were then centered on a canvas matching the pixel dimensions of the reference image; any areas extending beyond the canvas boundaries were cropped, and corresponding facial landmarks were adjusted accordingly. Expression images were aligned to the neutral facial expression by applying an affine transformation (incorporating translation, rotation, and scaling) estimated from all facial landmarks. This transformation was to normalize facial orientation and geometry.

A face mask was generated using 9 selected facial landmarks that define key regions such as the eyes, nose, mouth, and chin. Specifically, the landmarks corresponded to points 33 and 133 (left eye corners), 362 and 263 (right eye corners), 1 and 0 (nose bridge and tip), 13 and 14 (central points of the upper and lower lip), and 152 (chin), following the MediaPipe face mesh indexing scheme. The mask consisted of a rectangular upper half and an elliptical lower half, scaled with padding to cover the face region fully. To exclude hair and other non-facial areas, the region above the eyebrows was removed using eyebrow landmarks. The mask was then applied to isolate the facial region for further analysis (see Fig. 2b).

To reduce high-frequency differences caused by facial hair or skin texture, a slight Gaussian blur with a kernel size of 5 × 5 was first applied to both the reference face and the aligned expression face. The absolute difference between the two images was then computed and converted to grayscale (see Fig. 2c). The resulting difference image was smoothed using a Gaussian blur with a kernel size of 111 × 111 and scaled by a factor of 5 to enhance pixel intensities (see Fig. 2d,e). This processed difference image served as the basis for visualizing dynamic facial changes using a heatmap and for computing a facial symmetry score (see Fig. 2f,g). The heatmap was generated by applying a rainbow-like color map that enhances visual interpretation of facial movement by mapping low values to blue and high values to red.

A symmetry score was calculated by comparing the left and right facial halves within the masked area of the difference image. The right half was mirrored to align with the left side, and only overlapping, valid pixels were included in the comparison. The basic symmetry measure is the inverse of the mean absolute difference between the two halves:

1 $${S}_{mean}=1- \frac{1}{N}\sum_{i=1}^{N}\left|{L}_{i}-{R}_{i}\right|$$

where ${L}_{i}$ and ${R}_{i}$ are pixel intensities in the left and flipped right halves, and $N$ is the number of pixels. To penalize irregular asymmetries, the score is weighted by the variance of these differences:

2 $$S={S}_{mean}\times \left(1-min\left(\frac{{\sigma }^{2}}{{\sigma }_{max}^{2}}, 1\right)\right)$$

where ${\sigma }^{2}$ is the variance of the pixel differences, and ${\sigma }_{max}^{2}$ is a predefined maximum variance for normalization (set to 5000 in this study). The smaller ${\sigma }_{max}^{2}$, the faster the score decreases with increasing variance. The final symmetry score ranges from 0 (low symmetry) to 1 (high symmetry).

### Evaluation

All 405 facial image datasets from 198 patients were processed using the described image preprocessing and analysis pipeline. Subsequently, symmetry scores were computed and analyzed across different time points within each patient to assess changes throughout facial nerve recovery. To quantify progression, a robust trend analysis was applied to the individual symmetry scores of each patient. For patients with at least three measurements, a rolling median filter with a window size of three was applied to reduce the influence of outliers while preserving the overall trend. For patients with fewer measurements, the original values were used without filtering. The slope was then calculated using the Theil-Sen estimator, which is robust to outliers and small sample sizes. Based on the slope, trends were classified as improvement, deterioration, or no significant change. A conservative threshold of ± 0.001 was used to exclude minor fluctuations and identify only meaningful trends.

The Stennert Index values during movement were retrospectively extracted from patients’ clinical records at the time points of image acquisition when available. This index is a facial grading tool widely used in Germany in clinical routine^12^. It is assessed separately at rest and during voluntary facial movement, quantifying the severity of facial nerve palsy at rest on a scale from 0 (no asymmetry) to 4 (severe side differences), and during movement on a scale from 0 (all facial movements are normal) to 6 (total or near-total facial movement restrictions). In this study, only the values estimated during voluntary facial movement were considered. These values were documented as part of routine clinical care by multiple experienced clinicians, with each patient evaluated by a single examiner per time point. As no patient was assessed by more than one examiner at the same time point, and repeated ratings by the same examiner were not performed, intra- and inter-examiner reliability could not be formally assessed. The Stennert Index is a regional facial grading system comparable in structure and scoring logic to the Sunnybrook Facial Grading System, for which high intra- and interrater reliability has been demonstrated in previous studies^13–15^. While no dedicated reliability studies exist for the Stennert Index, it is reasonable to assume that, when applied by experienced raters, its reliability is comparable to that of the Sunnybrook system.

These extracted Stennert scores were then correlated with the image-derived symmetry scores to investigate the relationship between clinical assessments and objective measures of facial symmetry. Importantly, the clinical evaluations (Stennert Index) and the computational image analyses were conducted independently by different evaluators. The individuals responsible for the image processing and symmetry scoring were not involved in the clinical assessment of patients, thereby ensuring methodological independence and reducing potential confirmation bias. Spearman’s rank correlation coefficient was used to analyze this association, given the ordinal nature of the Stennert Index and the continuous nature of the symmetry scores. Statistical significance was determined based on corresponding *p* values (< 0.05 considered significant).

## Results

**Fig. 3:**
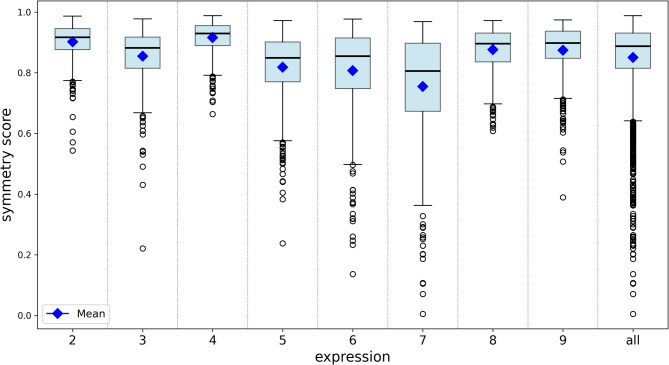
Boxplots of symmetry scores for all 405 datasets and 8 expression images (2–9), calculated relative to the reference image. The mean values are also indicated.

**Fig. 4:**
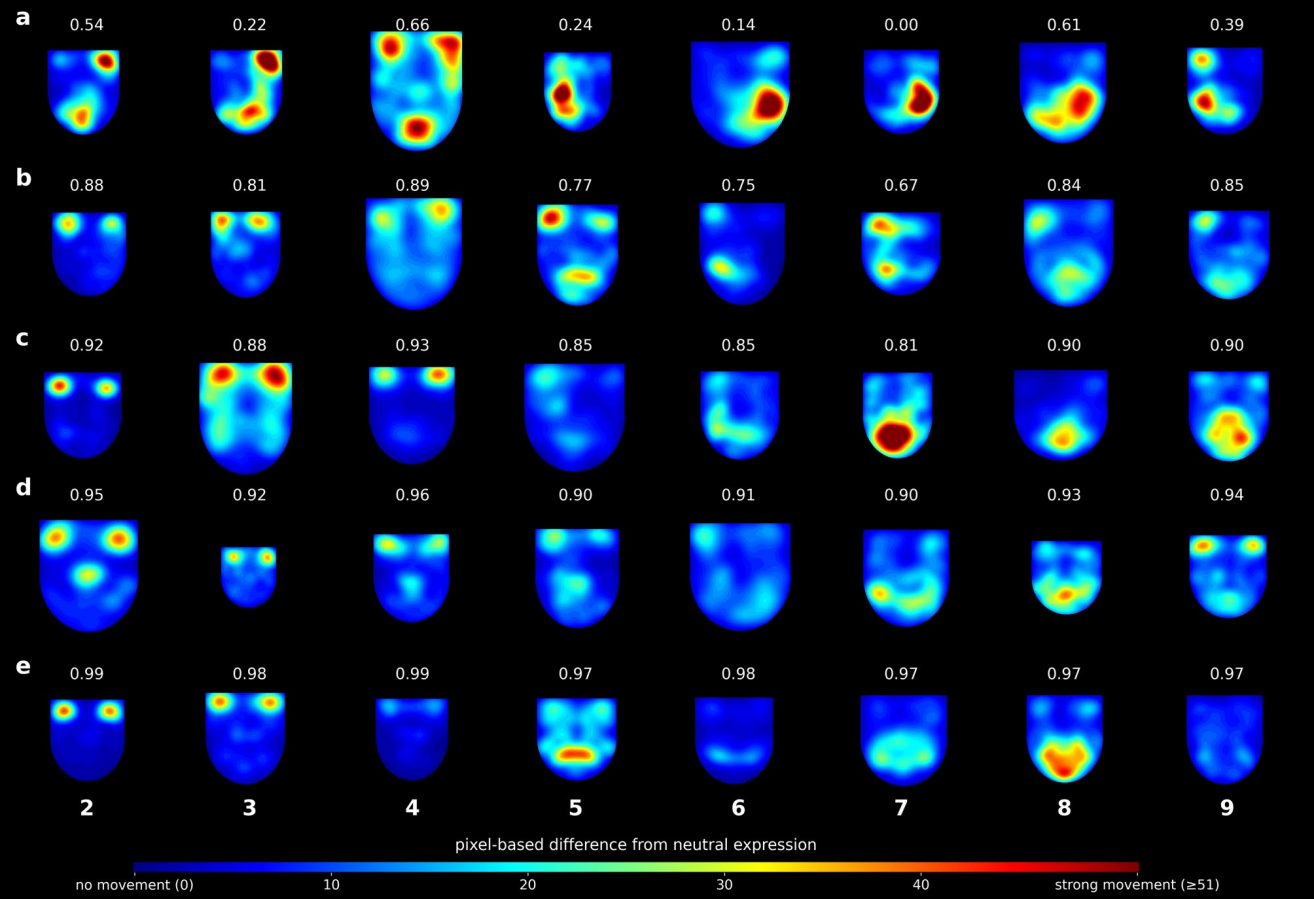
Example heatmaps illustrating different symmetry scores across 8 expression images (2–9) and 5 score levels: (**a**) minimum, (**b**) 25th percentile, (**c**) median, (**d**) 75th percentile, and (**e**) maximum symmetry scores. Pixel-based differences from the neutral expression are first smoothed using a Gaussian blur and then scaled by a factor of 5. Pixels with a difference of 51 or higher after smoothing appear at maximum color (red) in the heatmap (i.e., 51 × 5 = 255 on an 8-bit scale).

The method was applicable to all 405 datasets, with symmetry scores ranging from 0 to 0.99 (mean 0.85 ± 0.12), showing differences depending on the expression performed (see Table 1). Notably, expressions 7, 6, 5, and 3 exhibited a wide range of scores across the 405 datasets (see Fig. 3). Figure 4 displays an example of the heatmap for different symmetry scores. Even without showing the original photograph, the dynamics of movement, such as a mouth stretch with teeth showing (expression 7) or a closed-mouth stretch (expression 6), are visible, clearly indicating the side of facial palsy.

**Table 1:** Summary statistics of symmetry scores for each of the eight facial expressions (2–9) and overall (“all”) across all 405 datasets.

	Symmetry score of expressions								
	2	3	4	5	6	7	8	9	all
Mean	0.90	0.85	0.92	0.82	0.81	0.76	0.88	0.87	0.85
SD	0.06	0.09	0.05	0.12	0.15	0.18	0.07	0.09	0.12
Median	0.92	0.88	0.93	0.85	0.85	0.81	0.90	0.90	0.89
Max	0.99	0.98	0.99	0.97	0.98	0.97	0.97	0.97	0.99
Min	0.54	0.22	0.66	0.24	0.14	0.00	0.61	0.39	0.00

The table shows the maximum (max), minimum (min), mean, standard deviation (SD), and median symmetry scores.

The image-derived symmetry scores were further analyzed in relation to the Stennert Index scores, which were available for 266 datasets across all severity levels (0–6). Mean symmetry scores with standard deviations were calculated for each Stennert Index category and expression (see Table 2). A clear trend of decreasing symmetry scores with increasing facial palsy severity was observed across expressions, particularly for expressions 6 and 7, which showed the largest variability across severity levels. Spearman’s rank correlation analyses revealed a significant negative correlation between the Stennert Index and symmetry scores across all expressions (Spearman r = − 0.32 to − 0.66, *p* < 0.0001 for all, see Table 3), indicating that higher clinical severity was associated with lower symmetry scores. This demonstrates that the automated image-based symmetry analysis aligns well with clinical grading while providing continuous, objective quantification.

**Table 2:** Mean symmetry scores (± standard deviation) stratified by Stennert Index (0–6) across eight facial expressions (columns 2–9).

Stennert Index	n	Symmetry score of expressions							
		2	3	4	5	6	7	8	9
0	58	0.93 ± 0.05	0.91 ± 0.06	0.95 ± 0.03	0.89 ± 0.07	0.89 ± 0.08	0.89 ± 0.07	0.92 ± 0.04	0.91 ± 0.05
1	27	0.92 ± 0.05	0.89 ± 0.06	0.94 ± 0.03	0.86 ± 0.06	0.89 ± 0.07	0.86 ± 0.08	0.92 ± 0.04	0.92 ± 0.06
2	35	0.90 ± 0.07	0.88 ± 0.07	0.92 ± 0.06	0.85 ± 0.10	0.89 ± 0.06	0.84 ± 0.09	0.89 ± 0.05	0.90 ± 0.05
3	21	0.91 ± 0.04	0.85 ± 0.10	0.93 ± 0.04	0.82 ± 0.13	0.83 ± 0.10	0.74 ± 0.14	0.87 ± 0.07	0.88 ± 0.07
4	40	0.91 ± 0.05	0.87 ± 0.07	0.91 ± 0.05	0.81 ± 0.11	0.79 ± 0.14	0.74 ± 0.17	0.86 ± 0.08	0.87 ± 0.07
5	22	0.89 ± 0.07	0.83 ± 0.06	0.92 ± 0.04	0.78 ± 0.14	0.71 ± 0.21	0.63 ± 0.25	0.85 ± 0.09	0.85 ± 0.1
6	63	0.88 ± 0.07	0.81 ± 0.12	0.89 ± 0.06	0.76 ± 0.13	0.71 ± 0.15	0.60 ± 0.18	0.85 ± 0.07	0.85 ± 0.11

Higher symmetry scores indicate greater facial movement symmetry between the affected and unaffected side. A trend of decreasing symmetry scores with increasing Stennert Index can be observed, particularly in mouth-related expressions, reflecting the association between higher facial palsy severity and reduced symmetry. n indicates the number of datasets per Stennert Index category.

**Table 3:** Spearman’s rank correlation coefficients (r) and corresponding *p* values for the association between the Stennert Index and image-derived symmetry scores across eight facial expressions (2–9) in 266 datasets.

	Expressions							
	2	3	4	5	6	7	8	9
Spearman r	− 0.33	− 0.43	− 0.42	− 0.44	− 0.59	− 0.66	− 0.44	− 0.32
*p* value	*p* < 0.0001							

Negative correlation coefficients indicate that higher clinical severity (higher Stennert Index) is associated with lower symmetry scores. All correlations are statistically significant, confirming a robust inverse relationship between clinical grading and objective facial symmetry measures.

**Fig. 5:**
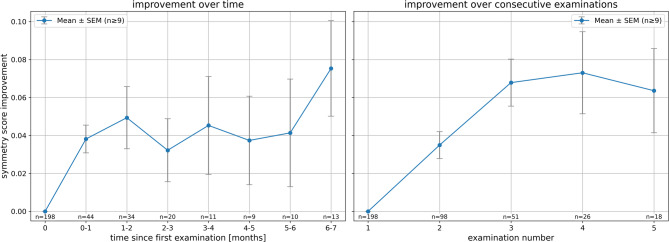
Improvement in symmetry score over time in 98 patients with at least two valid measurements. Both panels show mean changes relative to baseline with error bars representing the standard error of the mean (SEM) and include only groups with at least 9 observations. The left panel groups data into monthly time bins, while the right panel displays improvement across consecutive examinations. The number of patients contributing to each time bin or examination is indicated below the respective x-axes.

**Fig. 6:**
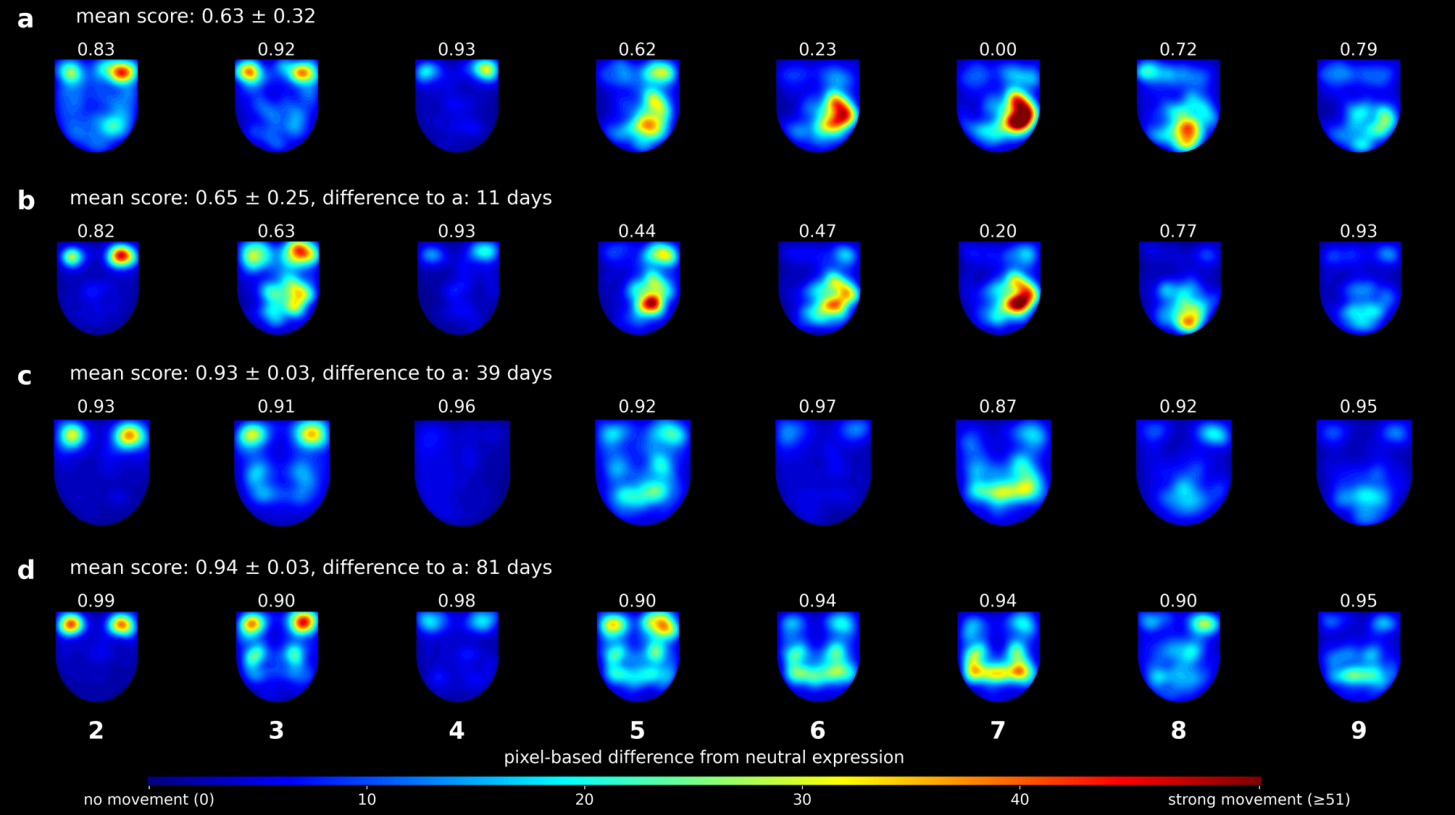
Example of heatmaps with symmetry scores for a single patient over time. Each row represents a different examination date, and each column corresponds to one of eight facial expression images (2–9). Scores above each image indicate the symmetry score for that specific expression. The figure illustrates how the score reflects the dynamic difference between the neutral reference and the performed expression. Limited or hesitant facial movements may lead to unexpectedly high scores, as exemplified in panel c4. Pixel-based differences from the neutral expression are first smoothed using a Gaussian blur and then scaled by a factor of 5. Pixels with a difference of 51 or higher after smoothing appear at maximum color (red) in the heatmap (i.e., 51 × 5 = 255 on an 8-bit scale).

Over the course of therapy, among 98 patients with at least two valid time points, 86% (84/98) showed an improvement in the symmetry score over time. In 14% of cases (14/98), the score either remained stable (2 cases) or deteriorated (12 cases). A tendency toward an early increase in symmetry score was observed during therapy (see Fig. 5), followed by notable individual variability in progression. As the evaluation timepoints were retrospective and not standardized, both the time intervals and examination numbers are only approximately comparable. However, for new patients, complete sets of recordings are usually obtained, making the therapy start and initial scores relatively reliable. Figure 6 shows an example of a single patient’s progression over time, illustrating that the dynamic change between the neutral facial expression and the performed expression influences the score. Limited movement, due to a hesitantly executed action, may result in a high score that might not be justified (see Fig. 6c4 for an example).

Where Stennert Index data were available, the trend between the symmetry scores and the Stennert movement scores was consistent in 85% (56/66) of cases. In 9% (6/66), the Stennert scores remained unchanged while the symmetry scores indicated improvement in 4 cases and deterioration in 2 cases. Only in 6% (4/66) of cases was there a discordant trend, which was minor in most instances. These discrepancies were due to substantial head movements during recording (2 cases) and insufficient facial movement during the expression (2 cases).

## Discussion

This study demonstrates that standardized photographic recordings can effectively generate privacy-compliant heatmaps visualizing dynamic facial movements and calculate an objective symmetry score in patients with PFP. The method proved robust across 405 datasets, capturing a broad range of symmetry scores and highlighting differences depending on the specific facial expressions performed. Notably, mouth-related expressions (expressions 6 and 7) were especially effective at revealing asymmetries, as reflected by their wide score distribution. The Stennert Index is a subjective clinical scoring system used to assess the severity of peripheral facial palsy by evaluating muscle strength, mobility, and facial asymmetry. In this study, the Stennert Index served as a reference standard to validate the image-based analysis results. The observed correlation between the symmetry score and the Stennert Index demonstrates that the automated method can deliver objective and quantitative assessments of facial function, which align well with clinical evaluations. Still photographs are a standard diagnostic tool to document the severity of the disease^16^. This means that such images are often available anyway. This underscores the potential of the image-based approach as a complementary tool to support and standardize clinical diagnostics. While the Stennert Index can in principle be assessed based on standardized facial photographs, the presence of the patient during the evaluation typically increases the reliability of the scoring. This is because it cannot be verified retrospectively whether the facial expressions were performed correctly or whether the photograph was taken at the optimal moment during movement. Table 4 provides an overview of the Stennert Index scoring system, with further methodological details available in Stennert et al.^12^.

**Table 4:** Stennert Index scoring system for facial nerve function.

Resting tone	Score	Motility	Score
Palpebral fissure difference	0: < 3 mm 1: ≥ 3 mm	Forehead wrinkling (> 50%)	0: possible 1: not possible
Ectropion	0: no 1: yes	Residual palpebral fissure (sleep)	0: no 1: yes
Flattened nasolabial fold (if present on healthy side)	0: no 1: yes	Residual palpebral fissure (max innervation)	0: no 1: yes
Mouth corner drooping	0: < 3 mm 1: ≥ 3 mm	Canine teeth visible (upper + lower)	0: visible 1: not visible
		Lateral incisor visible (upper)	0: visible 1: not visible
		Mouth corner shortening (vs healthy side)	0: ≥ 50% 1: < 50%
Paresis Index (Rest/Motility) 0–4/0–6			

Resting tone and motility are scored separately; higher scores reflect more severe dysfunction.

In this study, facial landmark detection using MediaPipe performed reliably even in patients with facial palsy (see Fig. 2a for an example), as demonstrated by the successful alignment and difference image calculation based on these landmarks. This conclusion is supported by another study^17^. Initial scaling based on interocular distance helped reduce image size and standardize resolution to the neutral reference, addressing dataset-specific size variations. However, this approach lacked precision, requiring an additional scaling step within the affine alignment using all landmarks to ensure consistent facial geometry and comparability. The facial mask was designed with a rectangular upper and elliptical lower half to match facial contours while excluding non-facial areas. Specifically, the region above the eyebrows was removed to avoid interference from hair movements, ensuring consistent isolation of the facial region. Nonetheless, facial hair remained within the mask and initially caused large pixel differences due to its differing grayscale intensity. Applying a slight Gaussian blur prior to computing the difference successfully mitigated this issue. After calculating the absolute difference between reference and expression images, a large Gaussian blur was applied to reduce measurement noise from slight misalignments or head movements, smoothing pixel-level variations while preserving overall movement patterns. The blurred difference image was then amplified to enhance the dynamic range of heatmaps and improve sensitivity in symmetry scoring. Both the blur size and amplification factor influence heatmap appearance and score distribution (see Supplementary Materials Figure S1). In this study, a uniform amplification was used for all expressions for consistency, but this parameter can be adjusted per expression to increase sensitivity and yield a broader, more informative range of symmetry scores.

Heatmaps enable clear visualization of facial dynamics while maintaining patient anonymity, as interpretation does not require the original images. Furthermore, heatmaps provide immediate and intuitive visual feedback for the therapist, potentially supporting therapy by helping patients understand and adjust facial movements in real time. Given that the method is based on lightweight MediaPipe landmark detection and straightforward image processing, it is well suited for mobile implementation. This opens the possibility of smartphone-based self-training tools, allowing patients to perform guided exercises at home with instant feedback on facial symmetry and movement patterns.

The symmetry score quantifies facial symmetry by comparing left and mirrored right regions within the mask. Variance weighting penalizes localized asymmetries, increasing sensitivity to subtle differences. The predefined maximum variance for normalization controls score sensitivity and range and can be tuned as needed (see Supplementary Materials Table S2). While the symmetry score depends on the quality of alignment and masking, it provides a simple, interpretable metric to track patient progress and evaluate therapy outcomes. Its computational simplicity enables real-time feedback, supporting patient engagement during rehabilitation. A limitation is that low facial movement can yield high symmetry scores, potentially masking functional deficits if the expression was not properly performed. However, this can be addressed by interpreting the accompanying heatmaps, which provide important visual context (see Fig. 6c6). Additionally, facial dynamics can be objectively measured by calculating the overall intensity of pixel differences between the reference face and the aligned expression face, enabling the identification of insufficient movements based on low movement intensity thresholds and the repetition of the exercise if needed.

In the literature, a recent systematic review^9^ demonstrates that there are still very few automated solutions available for assessing PFP. While some computerized tools show promise in providing objective and reproducible measurements, these approaches require further validation and resource investment before they can be widely adopted in clinical settings. Thus, manual clinical scales remain the standard for PFP assessment despite their subjective nature. Lee et al.^18^ automatically detect 68 facial landmarks using deep learning and quantify facial palsy by measuring landmark displacements during standardized facial expressions, while ensuring consistent head and jaw positioning to minimize distortions, thereby providing objective asymmetry scores that correlate well with the House-Brackmann and Sunnybrook grading systems. Similarly, Kim et al.^19^ employed automatic detection of 68 facial landmarks using the Emotrics software to evaluate facial palsy. Their approach involved a standardized photographic setup with a fixed head position to ensure consistency in measurements. Anping et al.^20^ automatically detected 68 facial landmarks on images of patients performing standardized facial movements and calculated angular and distance-based asymmetry indices within these images under neutral head positioning to objectively evaluate facial paralysis. Haase et al.^21^ used an automated Facial Action Coding System (FACS)-based analysis on standardized photographs to objectively assess facial asymmetry and track changes during therapy in PFP patients, offering a region-specific and fast assessment approach. Ten Harkel et al.^22^ developed an automated Sunnybrook grading system using Convolutional Neural Network (CNN)-based analysis of start and peak frames, achieving high agreement with clinicians and demonstrating feasibility for objective, reproducible facial palsy assessment in clinical and e-health settings. Raj et al.^23^ propose an automated, objective facial palsy grading method using deep feature extraction from pre-trained CNNs combined with linear regression, achieving an average grading error of around 11%, which improves consistency over subjective physician assessments and can support clinical treatment monitoring. More advanced and complex methods have also been explored. Recently, Büchner et al.^24^ introduced a dynamic 3D scanning method using multi-view landmarks and radial curves to quantify volumetric asymmetry in facial palsy. Their approach reduces manual effort and captures subtle changes during facial expressions, showing decreased asymmetry after therapy. Van Veen et al.^25^ employed 3D stereophotogrammetry and landmark analysis to quantify asymmetry, confirming the feasibility of objective assessment but highlighting the high cost and complexity of 3D systems for routine use. Similarly, Liu et al.^26^ applied infrared thermal imaging combined with machine learning techniques to analyze facial temperature asymmetry, achieving high accuracy in early detection of facial paralysis. However, such methods often require specialized equipment and expertise, limiting their accessibility.

In contrast, the proposed method uses standardized 2D photographs, providing a cost-effective, practical, and reproducible solution for objective facial symmetry analysis. Its lightweight computational requirements make it well suited for implementation on mobile devices, enabling accessible and real-time assessment in routine clinical and home settings. Unlike previous approaches that utilized fixed camera positioning and chin rests to ensure consistent head alignment^18,19^, the present study did not employ such fixation devices, based on the direct involvement and recollection of authors of this study who were clinically active at our institution during the image acquisition period. However, variations in camera angle and distance can artificially increase perceived dynamics and reduce comparability between sessions. Therefore, incorporating a standardized measurement setup with fixed camera positioning and optional chin support in future studies may further enhance consistency and reliability of automated assessments. Additionally, automated image capture and assignment to specific expressions, or automated extraction of neutral and expressive frames from short video recordings, could simplify the procedure by ensuring consistent input images without manual selection, making the assessment process more accessible and user-friendly for both clinical and home-based monitoring. Furthermore, future developments could focus on training an end-to-end deep learning model that directly processes paired neutral and expressive images to output heatmaps and symmetry scores without requiring intermediate landmark detection and alignment steps. This approach could further streamline and accelerate the assessment process, improving usability in routine clinical workflows and enabling robust. However, the development of such a powerful end-to-end model would require the collection of a large number of standardized, high-quality datasets to ensure sufficient variability and robustness for clinical application.

This study has several limitations. Data were collected retrospectively from a single hospital, which may limit generalizability. Moreover, this study serves as a proof of concept where parameters of signal processing were determined analytically. Further validation using larger datasets collected under standardized conditions is needed to confirm the method’s clinical utility. Additionally, since the method relies on the neutral facial expression as a reference for computing symmetry scores, cases with pronounced baseline asymmetry may influence the interpretation of the results. Nevertheless, as the analysis focuses on intraindividual changes between expressions and rest, it remains effective for capturing dynamic facial asymmetry. While the method quantifies facial movement independently of age-related anatomical differences, the small sample sizes in younger (< 20 years) and older (> 80 years) age groups limit the generalizability of the findings to these populations.

In conclusion, this study presents a novel, automated approach using standardized 2D photographs to objectively assess facial symmetry in patients with PFP. By visualizing dynamic facial changes through heatmaps and calculating a symmetry score, the method provides an interpretable and practical tool that could enhance clinical evaluation and patient monitoring. Future research should focus on optimizing score sensitivity, and validating the approach across a wider range of facial expressions and diverse patient populations.

## Supplementary Information

Below is the link to the electronic supplementary material.

Supplementary Material 1

## Data Availability

The datasets analyzed during the current study are not publicly available due to privacy concerns. However, all relevant data supporting the findings of this study are included in the article. For further inquiries or data requests, please contact the corresponding author.
