# Supplementary material for: Deep learning-based automatic facial symmetry scoring in peripheral facial palsy
Source: Sci Rep. 2025 Aug 27;15:31531. doi: 10.1038/s41598-025-17172-1 (PMC12391311; doi:10.1038/s41598-025-17172-1)
Supplement: Supplementary file 1 — Supplementary Material 1 [file 41598_2025_17172_MOESM1_ESM.pdf]

Deep Learning-Based Automatic Facial Symmetry Scoring in Peripheral Facial Palsy

Andreas Heinrich[1], Gerd Fabian Volk[2,3,4],
Christian Dobel[2,3,4], and Orlando Guntinas-Lichius[2,3,4]

[1]Department of Radiology, Jena University Hospital –
Friedrich Schiller University, Am Klinikum 1, 07747 Jena, Germany

[2]Department of Otorhinolaryngology, Jena University Hospital –
Friedrich Schiller University, Am Klinikum 1, 07747 Jena, Germany

[3]Facial Nerve Center Jena, Jena University Hospital –
Friedrich Schiller University, Am Klinikum 1, 07747 Jena, Germany

[4]Center for Rare Diseases, Jena University Hospital –
Friedrich Schiller University, Am Klinikum 1, 07747 Jena, Germany

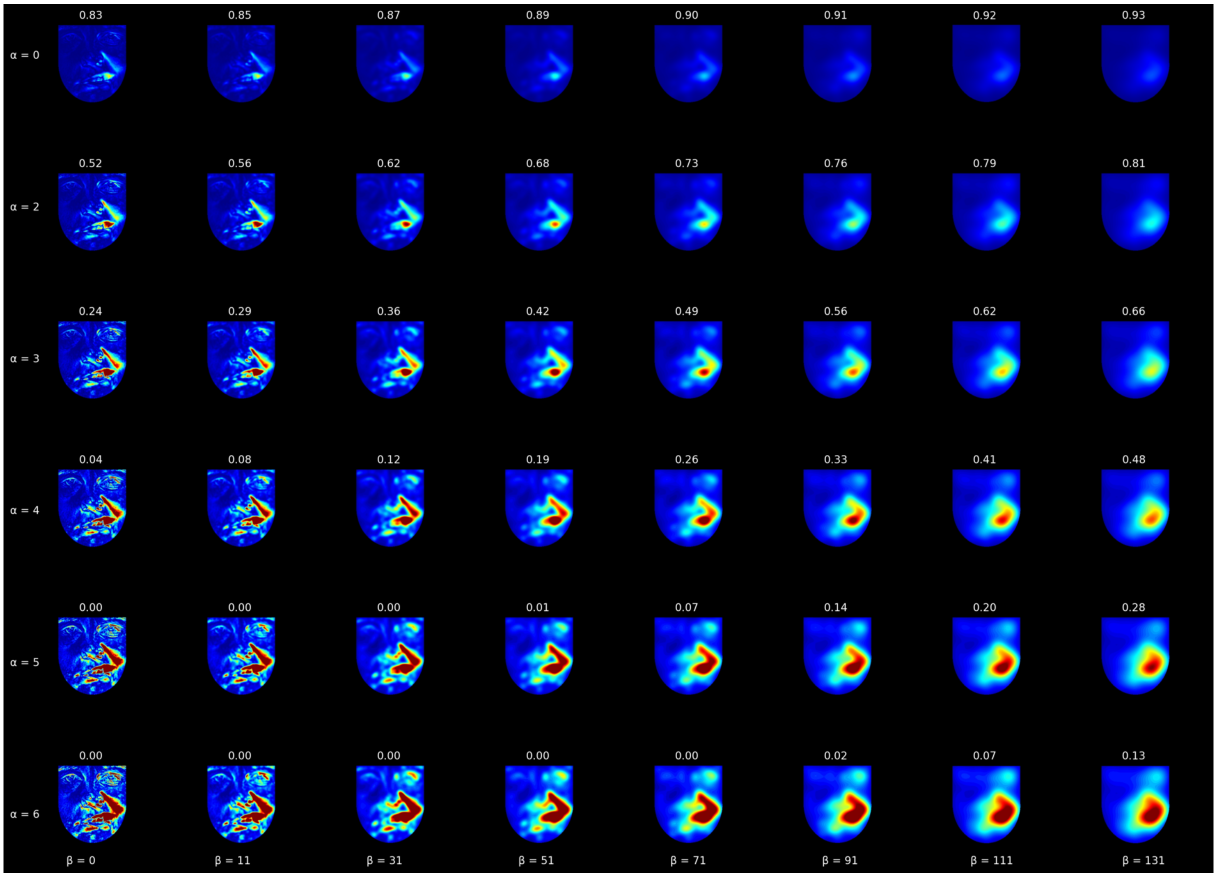

Figure S1 For the same patient shown in Figure 6 (panel b7), heatmaps with symmetry scores are displayed across different Gaussian blur parameters (β) and scaling factors (α) used to adjust pixel intensities.

σ_{max}^2	**symmetry score**							
	**2**	**3**	**4**	**5**	**6**	**7**	**8**	**9**
**1000**	0.36	0.00	0.85	0.00	0.00	0.00	0.20	0.84
**2000**	0.65	0.30	0.90	0.00	0.00	0.00	0.55	0.90
**3000**	0.75	0.48	0.92	0.23	0.25	0.00	0.67	0.91
**4000**	0.79	0.57	0.93	0.36	0.39	0.06	0.73	0.92
**5000**	0.82	0.63	0.93	0.44	0.47	0.20	0.77	0.93
**6000**	0.84	0.66	0.94	0.50	0.52	0.29	0.79	0.93
**7000**	0.86	0.69	0.94	0.53	0.56	0.36	0.81	0.94
**none**	0.94	0.84	0.95	0.76	0.79	0.75	0.91	0.95

Table S2 For the same patient shown in Figure 6b, the symmetry scores are presented for different values of the predefined maximum variance (σ_{max}^2) used for normalization. Each column corresponds to one of eight facial expression images (2–9). "None" indicates that only the basic symmetry score (see Equation 1) without variance weighting is provided.
